# Preclinical Evidence for the Interplay between Oxidative Stress and RIP1-Dependent Cell Death in Neurodegeneration: State of the Art and Possible Therapeutic Implications

**DOI:** 10.3390/antiox10101518

**Published:** 2021-09-24

**Authors:** Danuta Jantas, Władysław Lasoń

**Affiliations:** Maj Institute of Pharmacology Polish Academy of Sciences, Department of Experimental Neuroendocrinology, Smetna Street 12, PL 31-343 Krakow, Poland; lason@if-pan.krakow.pl

**Keywords:** neurodegenerative diseases, neuroprotection, necrostatin-1, necroptosis inhibitors, oxytosis, brain ischemia, TBI, SCI

## Abstract

Neurodegenerative diseases are the most frequent chronic, age-associated neurological pathologies having a major impact on the patient’s quality of life. Despite a heavy medical, social and economic burden they pose, no causative treatment is available for these diseases. Among the important pathogenic factors contributing to neuronal loss during neurodegeneration is elevated oxidative stress resulting from a disturbed balance between endogenous prooxidant and antioxidant systems. For many years, it was thought that increased oxidative stress was a cause of neuronal cell death executed via an apoptotic mechanism. However, in recent years it has been postulated that rather programmed necrosis (necroptosis) is the key form of neuronal death in the course of neurodegenerative diseases. Such assumption was supported by biochemical and morphological features of the dying cells as well as by the fact that various necroptosis inhibitors were neuroprotective in cellular and animal models of neurodegenerative diseases. In this review, we discuss the relationship between oxidative stress and RIP1-dependent necroptosis and apoptosis in the context of the pathomechanism of neurodegenerative disorders. Based on the published data mainly from cellular models of neurodegeneration linking oxidative stress and necroptosis, we postulate that administration of multipotential neuroprotectants with antioxidant and antinecroptotic properties may constitute an efficient pharmacotherapeutic strategy for the treatment of neurodegenerative diseases.

## 1. Introduction

Oxidative stress was originally conceptualized as “A disturbance in the prooxidant-antioxidant balance in favor of the former” [1]. More recently, this phenomenon has been defined as “An imbalance between oxidants and antioxidants in favor of the oxidants, leading to a disruption of redox signaling and control and/or molecular damage” [2]. A vast body of literature provided compelling evidence for the involvement of oxidative stress in the regulation of physiological and pathological processes in virtually all living systems [3,4]. Of these, the central nervous system (CNS) deserves special attention, especially because it shows a high demand for oxygen and relatively poor endogenous antioxidant defense [5,6]. The brain consumes about 20% of the body’s total oxygen due to its metabolic activity and high need for ATP (adenosine 5′-triphosphate). The brain is also rich in fatty acids, which are prone to oxidative damage in the process of lipid peroxidation, giving rise to unsaturated aldehydes, such as malondialdehyde (MDA), 4-hydroxynonenal (HNE) and acrolein [7]. Pathological effects of oxidative stress result from the excessive generation of highly reactive oxygen species (ROS), such as singlet oxygen (^1^O_2_), superoxide anion radical (O_2_^•−^), hydroxyl radical (HO^•^) and hydrogen peroxide (H_2_O_2_). Furthermore, superoxide anion radicals can combine with reactive nitrogen species (RNS), such as nitric oxide (NO), to generate the strong prooxidant peroxynitrite anion (ONOO^−^). The major endogenous producer of ROS, namely mitochondria, contains important elements of redox signaling, such as electron transport chain (ETC), NADPH oxidases (NOXs), xanthine oxidase and monoamine oxidases (MAOs). To maintain redox homeostasis, enzymatic (superoxide dismutase (SOD), glutathione peroxidase (GPx), glutathione reductase, catalase (CAT), thioredoxin (Trx) reductase) and non-enzymatic (glutathione (GSH), uric acid, bilirubin, coenzyme Q, α-lipoic acid, metallothionein, melatonin or metal-binding proteins) antioxidant systems are engaged [8,9]. When not balanced by endogenous antioxidants, free radicals bind to and damage the most vital cellular components, such as lipids, proteins, carbohydrates and nucleic acids [10]. Inefficient oxidative phosphorylation is the main source of ROS which in the next steps evoke changes in mitochondrial redox metabolism, phospholipid metabolism and proteolytic pathways. Moreover, oxidative stress produces a number of metabolic effects, such as a decrease in ATP level, reduction in GSH/GSSG (glutathione/oxidized GSH) ratio, disturbance of Ca^2+^ homeostasis, depolarization of cell membranes and dysfunction of ion channel functions and activation of poliADP-ribose synthesis [5,11].

It has been well established that mutations in mitochondrial DNA and oxidative stress contribute to aging, which is thought to be the greatest risk factor for neurodegenerative diseases (NDs) [12,13]. Despite various etiologies and symptoms, NDs possess some common biochemical and morphological features, i.e., cytoskeleton damage, protein aggregation, deficit of trophic factors and accompanying inflammatory processes. Oxidative stress is regarded as a pivotal pathogenic mechanism of neuronal death in both acute and chronic ND, although the exact causes of the latter remain unknown [5,6,8]. Thus, in Huntington’s disease (HD) the excitotoxicity-related degeneration of medium spiny neurons of the nucleus caudatus and putamen is associated with defects of mitochondrial ETC II, III and IV complexes resulting in oxidative stress and cell death [14]. Oxidative stress, which induces degeneration of dopaminergic neurons of the substantia nigra in Parkinson’s disease (PD) is accompanied by a decrease in detoxification enzymes (GSH), increase in Fe^2+^ and autoxidation of dopamine, enhanced metabolism of dopamine via MAO B, microglia activation, enhanced release of glutamate and NO production, disturbed Ca^2+^ homeostasis and defect of mitochondrial ETC complex I [15,16]. Amyotrophic Lateral Sclerosis (ALS) is characterized by selective degeneration of motor neurons, which is linked to excitotoxicity (defect of AMPA receptor function), increase in Ca^2+^ influx, decrease in excitatory amino acid transporter 2 (EAAT-2) levels, mutation of SOD1 and defect of mitochondrial ETC complex IV, ultimately leading to oxidative stress and motor neuron death [17]. In Alzheimer’s disease (AD), the degeneration of cortical and limbic structures is associated with mutation of APP (Amyloid-beta precursor protein) and presenilin-1, and with defect of ETC complex IV [18,19]. While in chronic ND there is a rather moderate increase in the level of intracellular reactive species evoked by gradual dysfunctions of various organelles, in acute CNS damage, it is a rapid process with high generation of ROS or RNS. In the case of ischemic stroke, the obstruction of blood supply to the brain evokes metabolic disturbances resulting in increased oxidative stress and inflammation. These events lead to non-selective death of neuronal and other types of cells, which could be further exacerbated during reperfusion [20,21]. In hemorrhagic stroke, a less common but also life-threatening form of stroke, the iron derived from blood, which enters the brain tissue, initiates a cascade of pathophysiological changes, such as depolarization, excitotoxicity, oxidative stress, disrupted ionic homeostasis, cell edema, inflammatory response and secondary BBB (blood-brain barrier) disruption [22]. Traumatic brain injuries (TBI) or spinal cord injuries (SCI) cause metabolic and ionic imbalance, which eventually leads to excessive production of ROS [23,24]. It should be stressed that both clinical and preclinical studies show that acute and chronic ND are characterized by higher levels of oxidative stress biomarkers and by lower levels of antioxidant defense biomarkers in the brain and peripheral tissues [6,8,25]. 

At the molecular level, oxidative stress appears to participate in both unregulated (necrosis) and regulated (apoptosis, necroptosis, ferroptosis, pyroptosis, etc.) forms of neuronal death [26]. Interestingly, in contrast to the cell-damaging effect of ROS at high concentrations, free radicals at low concentrations play a physiological role in cell signaling. Moreover, low concentrations of ROS activate cell survival signaling pathways: UPR (unfolded protein response) and Nrf2 (nuclear factor erythroid 2–related factor 2), whereas in high concentrations they activate programmed cell death signaling pathways, such as apoptosis or necroptosis [27,28]. Prevailing evidence suggests that in contrast to apoptosis, which is essential for the development and survival of multicellular organisms, necroptosis may be the key form of neuronal death in the course of ND [29,30,31]. Surprisingly, the relationship between oxidative stress and necroptosis in the context of the pathomechanism of ND has not been reviewed in detail so far. Therefore, in this paper, we aimed to collect and discuss preclinical evidence for an interplay between oxidative stress and necroptosis obtained in cellular and animal models of acute and chronic neurodegeneration.

## 2. Mechanisms of Necroptosis 

**Figure 1:**
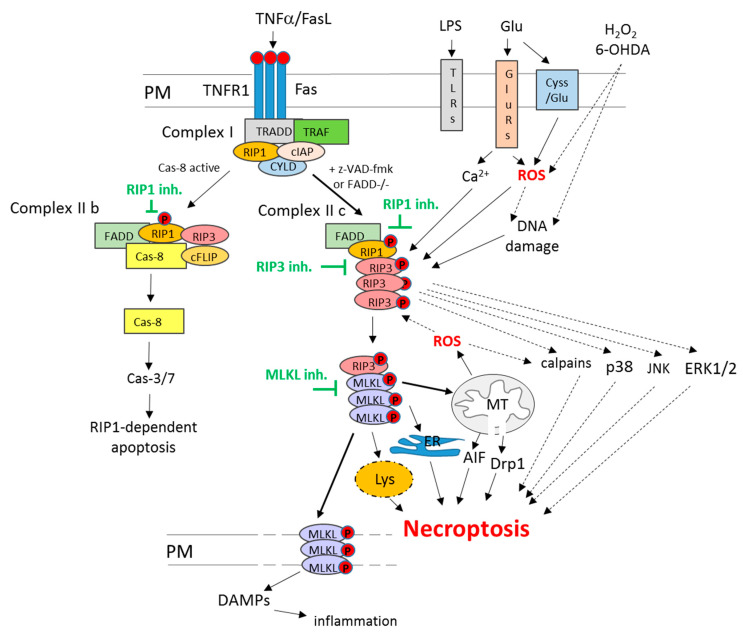
Mechanisms of RIP1-dependent necroptosis and apoptosis. The RIP1-dependent cell death in the classical way can be induced via activation of the death receptor pathway. In the first step, TNFα or FasL, via binding to the respective receptor (TNFR1 or Fas), triggers complex I assembly (TNFR1, TRADD, RIP1, TRAF2, cIAPs and CYLD). Under unavailability of TRADD or cIAPs, complex II b is formed (RIP1, RIP3, FADD and caspase 8), which induces RIP1-dependent apoptosis. Another scenario is initiated when caspase-8 or FADD are deficient then complex II c (necrosome) built from RIP1-RIP3 is formed. Subsequently, it phosphorylates and recruits MLKL, which oligomerize in plasma or other intracellular organs (mitochondria, ER, lysosomes) membranes, leading to their damage and cell death via necroptosis. Moreover, plasma membrane perforation evokes the release of DAMPs, which induces inflammatory response in surrounding tissue. Among other inducers of necroptosis, the following can be listed: LPS, TLRs, ROS, DNA damage, excitotoxicity and calcium overload. Beyond MLKL, among other postulated executioners of necroptosis, ROS, AIF, Drp-1, JNK, p38, ERK1/2, calpains and lysosome enzymes can be mentioned. Abbreviations: AIF—Apoptosis-Inducing Factor; cas-3/7—caspase 3/7; cas-8—caspase 8; cIAPs—cellular inhibitor of apoptosis proteins; CYLD—cylindromatosis; DAMPs—Damage-Associated Molecular Patterns; Drp1—Dynamin-related protein; ER—endoplasmic reticulum; ERK1/2—extracellular signal-regulated kinase 1/2; Fas—death receptor Fas; FasL—Fas ligand; Glu—glutamate; GluRs—glutamate receptors; inh.—inhibitors; JNK—c-JUN N-terminal kinase; LPS—liposaccharide; Lys—lysosomes; MLKL—mixed lineage kinase domain-like protein; PM—plasma membrane; RIP1—receptor-interacting protein kinase 1; RIP3—receptor-interacting protein kinase 3; ROS—reactive oxygen species; TLRs—Toll-like receptors; TNFα—tumor necrosis factor alpha; TNFR1—TNF receptor 1; TRADD—TNFR1-associated death domain; TRAF2—TNFR-associated factor 2.

Among various forms of non-apoptotic programmed cell death pathways, necroptosis (or necroptotic cell death) is the most widely studied so far. The morphological features that all these cell death routes have in common are unspecific necrotic changes, including the loss of plasma membrane integrity, swelling of cellular organelles and lack of typical nuclear fragmentation [32]. The distinction between various forms of regulated necrosis, such as necroptosis, ferroptosis, parthanatos or cyclophilin d-dependent necrosis, could be done on the basis of specific intracellular mechanisms involved [26,30,33,34]. In the case of necroptosis, such key players include two members of receptor-interacting serine/threonine-protein kinase (RIP) family, RIP1 and RIP3 (known also as RIPK1 and RIPK3). Classically, necroptosis is activated via activation of death receptors (TNFR1 or Fas) under caspase inhibition (Figure 1), as shown by the pioneering work of Degterev’s research group in the first decade of the 21st century and was described as an alternative form of programmed cell death when apoptosis was blocked. In this scenario, TNFα or FasL in U937 cell line in the presence of a caspases inhibitor (z-VAD-fmk) or in FADD (Fas-associated via death domain)-deficient Jurkat T cells via binding to TNFR1 and Fas receptor-induced cell damage without apoptotic signs but showing necrotic morphology (mitochondrial collapse and increase in the number of necrotic nuclei) which was reduced by necroptosis inhibitor, necrostatin 1 (Nec-1) [35]. Later on, the same group showed that RIP1 was essential for necroptosis execution, and its inhibition by Nec-1 was mechanistically responsible for cytoprotective action of the latter compound [36]. Other research groups evidenced the importance of the interaction between RIP1 and RIP3 in necroptosis execution, which, by forming necrosome complex, led to activation of the pseudokinase MLKL (mixed lineage kinase-like protein kinase) [37,38]. MLKL forms oligomers that translocate to the plasma membrane and cause its permeabilization leading to cell damage (Figure 1). This promotes the release of DAMPs (Damage-Associated Molecular Patterns), such as HMGB1 (high-mobility group box 1 protein) or mitochondrial DNA, which in vivo induce an immune response and inflammation [30,31,32,39]. 

The portfolio of factors that have been reported to induce necroptosis is wide and includes bacteria and viruses, LPS (liposaccharide) and TLRs (Toll-like receptors), increased oxidative stress, genotoxic stress, excitotoxicity and calcium overload (Figure 1). However, it is still not fully recognized how mechanistically they contribute to the activation of this type of cell death [30,40,41]. While the upstream regulators (deubiquitinase CYLD, caspase-8, FADD) and mediators (RIP1, RIP3, MLKL) of necroptosis are quite well described (Figure 1), the executioners of necroptosis and their interactions with other cell death programs are only partially recognized [34,42]. Beyond MLKL, necroptosis has been reported to engage the activation of JNK (c-JUN N-terminal kinase), p38 and ERK1/2 (extracellular signal-regulated kinase 1/2) signaling, AIF (Apoptosis Inducing Factor) nuclear translocation, activation of calpains, increase in ROS level and stimulation of sphingomyelinases (Figure 1). Moreover, necroptosis has been associated with the release of cytotoxic factors from lysosomes (hydrolases, proteases) and mitochondria (mitochondrial phosphoglycerate mutase/protein phosphatase PGMA5, Dynamin-related protein 1 (Drp1)), which also contribute to cell demise [43,44,45,46,47,48,49].

It should be noted that necroptosis could be also executed in a non-classical way without the involvement of RIP1 (RIP1-independent necroptosis) [50,51]. The molecular mechanisms and cellular signaling pathways involved in necroptosis are quite complex, and some steps can be shared with other types of programmed cell death pathways (e.g., extracellular apoptosis), which are often regulated in a context- and cell-type-dependent manner [32,33,34,39,52]. Apart from being a pathological factor in acute injuries of various organs (brain, heart, lung, kidney, liver, intestine and pancreas) and being associated with the pathogenesis of various ND [29,39], necroptosis plays also an important physiological role during development and adulthood. Of note, it is also involved in the regulation of the immune system and inflammatory response and participates in the elimination of infected cells in host defense [31,53]. Thus, great caution should be exercised when using necroptosis inhibitors for the management of age-associated ND, since their chronic administration could evoke undesired effects in immune system functioning [20,54]. Nevertheless, some experimental data from studies with transgenic models and enzymatic inhibitors suggest that RIP1, RIP3 or MLKL could be targeted for the treatment of various inflammatory, degenerative and infectious disorders including also acute and chronic neurodegenerative diseases [20,30,40,54].

## 3. Necroptosis Inhibitors

### 3.1. RIP1-Targeted Inhibitors 

The first-generation necroptosis inhibitors are named necrostatins and belong to (5-(1H-Indol-3-ylmethyl)-2-thiohydantoins or 5-(1H-indol-3-ylmethyl)hydantoins. They were selected from the library of ~15,000 compounds in a phenotypic screen employing human monocytic U937 cell line exposed to TNFα+z-VAD-fmk [35] or discovered by SAR (structure–activity relationship) study in necroptotic cell death model induced by TNFα in FADD-deficient Jurkat T cells [55]. These compounds, with the best-characterized Nec-1 (5-(1H-Indol-3-ylmethyl)-(2-thio-3-methyl) hydantoin, methyl-thiohydantoin-tryptophan), act via stabilization of an inactive conformation of the RIP1 kinase domain and in this way inhibit RIP1-dependent necroptosis [36]. Since these compounds are characterized by low metabolic stability, low solubility and low specificity, more stable and specific analogues of Nec-1 have been proposed, such as Nec-1s (Nec-1 stable, 7-Cl-O-Nec-1, Nec-2, 5-((7-chloro-1H-indol-3-yl)mevthyl)-3-methyl-2,4-imidazolidinedione), Nec-3, Nec-5 or Nec-7 [36,56,57]. Among them, Nec-1s showed significantly improved pharmacokinetic properties (better stability and solubility) and pharmacodynamics (higher affinity for RIP1; better antinecroptotic acivity; lack of effect on indoleamine 2,3-dioxygenase (IDO)), and therefore have been recommended especially for in vivo studies [58,59]. In parallel, an inactive compound Nec-1i (Nec-1 inactive, 5-(Indol-3-ylmethyl)-2-thiohydantoin), which inhibits IDO but not RIP1, was selected and recommended as a negative control for the mechanistic studies on necroptosis [36,58]. However, Nec-1 and other allosteric RIP1 inhibitors show an ability to inhibit RIP1-dependent apoptosis, which could at least in part contribute to their neuroprotective effects [31]. Moreover, other off-targets of Nec-1 such as RIP3, PAK1 (p21-activated kinase-1) or PKAcα (cAMP-dependent protein kinase catalytic subunit alpha) should also be taken under consideration when discussing the mechanisms of Nec-1-mediated neuroprotection [60,61,62]. 

The description of the crystal structure of RIP1 in 2013 facilitated further rational design and development of RIP1 inhibitors [63]. Next, screens performed in the clinical kinase library identified multi-targeted tyrosine kinase inhibitors, such as pazopanib, used in the treatment of advanced renal cell carcinoma and soft tissue sarcoma, and ponatinib, an antileukemic drug, as moderate inhibitors of RIP1, with similar or better potency than Nec-1s [64]. Ponatinib was shown to also bind to RIP3; thus, a series of ponatinib-Nec-1 (PN) hybrids was created by the inclusion of a fragment of this anti-cancer drug [60]. A representative of this group, compound PN10, is characterized by ~20-fold greater antinecroptotic activity and high RIP1-selectivity, being also efficient at a low dose (0.4 mg/kg) in the mouse SIRS (systemic inflammatory response syndrome) model [60]. Another class of RIP1-inhibitors (1-aminoisoquinolines, pyrrolo[2, 3-b]pyridines and furo[2,3-d]pyrimidines) were proposed by a GlaxoSmithKline (GSK) group [65,66]. For example, compound Cpd27 showed a potent anti-RIP1-kinase activity and antinecroptotic activity and the ability to block the TNFα-induced mortality in a mouse SIRS model at a dose of 20 mg/kg [65]. Another compound developed by this group GSK’963 (2,2-Dimethyl-1-(5(*S*)-phenyl-4,5-dihydro-pyrazol-1-yl)-propan-1-one) showed a high level of necroptosis inhibition in both murine and human cells with superior to Nec-1 effects. Moreover, this agent was highly potent in the RIP1 binding assay and was protective in vivo in lethal shock induced by TNFα at a dose of 2 mg/kg [66]. Although more than 40 patent applications on small-molecule drug candidates targeting RIP1 have been published since 2016, to date, only a few of this class of inhibitors have been or are already being tested in the clinic, e.g., in rheumatoid arthritis, psoriasis, ulcerative colitis, neurodegenerative diseases (AD or ALS) and severe COVID-19 lung disease [67]. Since outcomes of some of these trials were unsatisfactory, there is still a need for optimization of clinical settings, dosing and therapeutic indications for RIP1 inhibition as well as for further development of drugs from this class of small molecules [67].

### 3.2. RIP3-Targeted Inhibitors

In order to study the mechanisms of necroptosis and to look for potential clinically relevant drugs that target lower steps in necroptosis machinery, some small molecule inhibitors have been designed [30,68]. For example, the GSK team has developed a series of GSK compounds targeting human RIP3; however, some of them (GSK’843 and GSK’872) at higher concentrations induced RIP1-dependent apoptosis, and it has been postulated that RIP3 holds both necroptosis and apoptosis in balance through a ripoptosome-like platform. Furthermore, the compound GSK’840, highly active in human cell cultures, was not active in cellular murine models, precluding its verification in in vivo animal models [69]. Another representative of efficient RIP3 inhibitors, namely GW’39B, displayed activity in a number of necroptosis assays in human and murine cells. Finally, a screen of the clinical kinase inhibitors showed that dabrafenib, a B-RafV600E inhibitor used in the treatment of melanoma, possesses antinecroptotic activity, targeting RIP3, and besides cellular models, it was also effective in vivo against acetaminophen-induced liver injury in mice [70]. Since RIP3 could play differential roles in various cell phenotypes, as a double-edged sword effect [71,72], the chronic inhibition of this target required for treatment of chronic neurodegenerative diseases could be problematic.

### 3.3. MLKL-Targeted Inhibitors

The first reported MLKL inhibitor, i.e., necrosulfonamide (NSA, (E)-*N*-(4-(*N*-(4,6-dimethylpyrimidin-2-yl)sulfamoyl)phenyl)-3-(5-nitrothiophene-2-yl)acrylamide), has been often used as a research tool to study the mechanisms of necroptosis in human cellular models. However, due to its low activity against murine MLKL, it has not been studied in animal models. Thus, a new class of MLKL inhibitors has been proposed. For example, “compound 1” (GW806742X, SYN-1215) was efficient in blocking MLKL upon RIP3-mediated phosphorylation, thus preventing the MLKL oligomerization and translocation. As some doubts about its specificity (also affinity for RIP1) have been raised, its usefulness as a specific research tool to investigate MLKL’s role in necroptosis has been questioned. Since most MLKL inhibitors are not active in murine experimental models, their in vivo potency has not been confirmed yet [30,68].

Although various derivatives of Nec-1 as well as other inhibitors of necroptosis with higher specificity and antinecroptotic efficiency than Nec-1 have been synthesized and investigated in screening necroptotic cellular models, most of them still showed unfavorable pharmacokinetic profiles and only some of them have been tested in animal studies. Thus Nec-1 remains the most often used experimental tool to study the role of necroptosis in various physiological and pathological conditions [30,40,42,54].

## 4. In Vitro Evidence for the Interplay between Oxidative Stress and Necroptosis

### 4.1. Neuroprotective Effects of Necroptosis Inhibitors in Cellular Oxidative Stress Models

Excessive oxidative stress in in vitro settings is frequently modeled with the addition of hydrogen peroxide (H_2_O_2_) and/or BSO (buthionine sulfoximine, which depletes endogenous GSH). In such models, by using necroptosis inhibitors, mainly Nec-1, researchers obtained evidence that oxidative stress inducers evoke neuronal cell death via activation of necroptosis (Table 1). In mouse hippocampal HT-22 cells, Nec-1 completely prevented the cell damage induced by BSO, and this effect was connected with an increased GSH level [73]. In other neuronal-like cell models, human neuroblastoma SK-N-SH cells, it was found that H_2_O_2_ in the presence of GSH depletion (BSO) induced necroptotic cell death, which was completely blocked by Nec-1 via mechanisms engaging the inhibition of p38 signaling and alleviation of ROS production [74]. In the next study, Jantas et al. [46] showed that pretreatment with Nec-1 but not z-VAD-fmk partially attenuated the H_2_O_2_-induced cell damage in HT-22 cells. Moreover, Nec-1 attenuated the cell death induced by H_2_O_2_ in human neuroblastoma SH-SY5Y cells, and this effect was greater in undifferentiated (UN-) cells when compared to retinoic acid (RA)-differentiated ones suggesting some common mechanisms for a pro-survival effect of Nec-1 and RA [46]. The protection mediated by Nec-1 in this cellular model of oxidative stress in UN- and RA-SH-SY5Y cells was not connected with the inhibition of caspase-3, calpains or AIF translocation but was associated with the inhibition of the lysosomal protease, cathepsin D activity. Moreover, Nec-1 pretreatment completely attenuated the H_2_O_2_-evoked neurite shortening in UN- and RA-SH-SY5Y cells [46]. Interestingly, the protective effect of Nec-1 against H_2_O_2_-induced cell damage in HT-22 cells was blocked by pan-caspase inhibitor z-VAD-fmk, whereas a caspase-3 inhibitor (Ac-DEVD-CHO) did not affect the range of Nec-1-mediated protection in SH-SY5Y cells, which pointed to a cell type-specific interplay between oxidative stress and necroptosis, and between oxidative stress and apoptosis [46].

Another cellular model of oxidative stress in neuronal-like cells could be achieved by glutamate (Glu) exposure [75]. It is worth underlining that the mechanisms of Glu-induced cell damage in neuronal-like cell models (such as immortalized mouse hippocampal HT-22 cell line, immortalized mouse RGC-5 cells (retinal ganglion cell line) or human neuroblastoma SH-SY5Y cells), which possess glycolytic phenotype, differ from those found in primary neuronal cell cultures, where the latter operate mainly by oxidative phosphorylation. It has been shown that exposure of the above cells to high concentrations of Glu (>3 mM) evokes oxytosis, which is linked with reversal of the action of the cysteine (CySS)/Glu antiporter, inhibition of CySS uptake and decrease in intracellular glutathione (GSH) level, which leads to increased ROS level and oxidation of intracellular macromolecules (proteins, lipids and DNA) [75]. Furthermore, various cell death programs could be engaged in the execution of Glu-induced oxytosis, such as caspase-3 and/or caspase-3-independent (AIF-dependent) apoptosis or necroptosis [75,76]. Several reports demonstrated that Nec-1 could be highly protective against Glu-evoked oxytosis. First, Xu et al. [73] reported that Nec-1 was protective against the Glu-evoked cell damage in HT-22 cells which was connected with inhibition of AIF nuclear translocation, prevention of GSH reduction and attenuation of Glu-stimulated increase in mitochondrial BNIP3 (Bcl-2/adenovirus E1B 19 kDa-interacting protein 3) level. In another study, Zhang et al. [77] demonstrated a critical role of necroptosis and MAPK/ERK1/2 activation in the Glu-induced oxytosis in HT-22 cells. Inhibitors of ERK1/2 and Nec-1 but not Nec-1i almost completely prevented the Glu-evoked cell damage, which was associated with attenuation of phospho-ERK1/2 level [77]. Recently, Park et al. [76] found that N-acetyl-l-cysteine (NAC) and Nec-1, but not the caspase inhibitor z-VAD-fmk, completely prevented the Glu-induced toxicity in HT-22 cells. This is in line with the results obtained in our studies [46], which demonstrated that Nec-1 and NAC but not z-VAD-fmk reduced the Glu-toxicity in HT-22 cells. However, z-VAD-fmk, when given concomitantly with Nec-1, blocked the neuroprotective effects of the latter (in a similar way as it did in the H_2_O_2_ cell damage model) suggesting the interplay between apoptotic and necroptotic machinery [46]. Recently, Gonzales et al. [78] revealed that Nec-1 partially attenuated the Glu (160 mM)-evoked cell damage in RA-differentiated SH-SY5Y cells, and it was associated with a reduction in intracellular ROS level and caspase-3 activity. In that study, also NAC and iron chelator deferoxamine (DFO) were protective suggesting an interplay between necroptosis and ferroptosis in Glu-mediated oxytosis at least in human neuroblastoma cells [78]. Additionally, in mouse RGC-5 cells, a cellular model of glaucoma where oxidative stress significantly contributes to its pathogenesis, pretreatment with Nec-1 and NAC but not Nec-1i or z-VAD-fmk partially attenuated the cell damage evoked by Glu+BSO [79,80]. Moreover, in this study, neuroprotective effects were found for aspirin derivatives (ACS1 and ASC14) which attenuated Glu+BSO-induced ROS formation, and their protection range was similar to Nec-1 effects, suggesting that the involvement of necroptosis inhibition in the mechanism of action ACS1 and ASC14 [80]. Oxidative damage is also implicated in white matter injury, and oligodendrocyte precursor cells (OPCs) are highly susceptible to various forms of oxidative stress. The study by Kim et al. [81] has shown that co-treatment with Nec-1 completely prevented cell damage induced by arachidonic acid, BSO or cysteine deprivation but not by H_2_O_2_ in 7–9 DIV OPCs. These protective effects of Nec-1 were accompanied with alleviation of ROS production and inhibition of JNK signaling [81].

The data presented above obtained in various oxidative stress cellular models in neuronal and other brain cell types where Nec-1 was neuroprotective, directly support the hypothesis that oxidative stress could be an inducer of necroptosis and the protection of similar magnitude can be achieved by antioxidants (e.g., NAC) or necroptosis inhibitors (e.g., Nec-1).

### 4.2. Neuroprotective Effects of Necroptosis Inhibitors in In Vitro Excitotoxicity Models

Glu is the main excitatory neurotransmitter in CNS playing a crucial role during development and adulthood. However, an excessive extracellular amount of Glu could be detrimental to neurons due to evoking the process of excitotoxicity (toxicity resulting from uncontrolled continuous depolarization), which is postulated to be involved in the pathogenesis of various acute and chronic brain diseases [75,82]. During excitotoxicity, the overactivation of ionotropic Glu receptors (mainly *N*-methyl-d-aspartate (NMDA) receptors) leads to a rise in intracellular Ca^2+^ levels, upregulation of nNOS (neuronal nitric oxide synthase), mitochondrial collapse, increase in ROS level, ER (endoplasmic reticulum) stress and release of lysosomal enzymes. In neuronal cellular models, depending on the concentration of excitatory agents (Glu, NMDA or kainate) and stage of cell development, apoptotic and/or non-apoptotic forms of cell death programs could be initiated [75]. The first evidence that necroptosis contributes to the NMDA-induced excitotoxicity was provided by Li et al. [83]. The authors found that Nec-1 moderately inhibited the NMDA-induced cell damage in 10–12 DIV primary cortical neurons, which was associated with partial attenuation of NMDA-evoked rise in intracellular Ca^2+^ level [83]. Interestingly, a study by Hernandez et al. [84] demonstrated that excitotoxicity induced by Glu could lead to neuronal soma apoptosis, but axons degenerated by induction of necroptosis. In such a model of 7–15 DIV hippocampal neurons, the axonal degeneration induced by Glu was completely prevented by Nec-1 via mechanisms associated with attenuation of intracellular Ca^2+^ level and improvement of axonal mitochondrial function. These data demonstrated differential programmed cell death mechanisms in two cellular compartments under the same excitotoxic stimulus [84] and justify combined treatment with antiapoptotic and antinecroptotic compounds to combat neurodegenerative processes. The two above-mentioned examples of studies showing neuroprotection provided by Nec-1 against NMDA- and Glu-evoked neuronal cell death (Table 1) indirectly point to possible ROS involvement based on known sequential mechanisms of excitotoxicity [75]. However, this needs to be functionally verified in future studies by measurement of necroptosis markers and/or using cells with depleted necroptotic machinery.

### 4.3. Neuroprotective Effects of Necroptosis Inhibitors in In Vitro Ischemia/Hypoxia Models

Global brain ischemia (stroke) can be modeled in cellular systems using primary neuronal or neuronal-glia cell cultures exposed to oxygen-glucose deprivation (OGD). This procedure leads to the breakdown of cellular integrity by ionic imbalance, energetic collapse, excitotoxicity and increased ROS production. The neuronal cell damage can be executed via rapid nonspecific necrosis (cell lysis) or by programmed cell death pathways (apoptosis or necroptosis), depending on the duration of the OGD and recovery (re-oxygenation) time (OGD/R) [21]. A large body of evidence shows that necroptosis could be involved in OGD/R-evoked neuronal cell damage since Nec-1 or other necroptosis inhibitors have been shown to be at least partially neuroprotective in such models (Table 1). It was confirmed in various neuronal cell models including immature or mature primary neuronal cell cultures [85,86,87,88,89,90], PC12 cells [88] or RGC-5 cells [91,92], although parameters of oxidative stress have not been measured in these studies. Apart from neurons, other cell types, such as astrocytes, oligodendrocytes or microglia cells could also be partially protected by Nec-1 against the OGD/R-evoked cell death [89,93,94,95,96]. Moreover, DTIO (5-(3′,5′-dimethoxybenzal)-2-thio-imidazole-4-ketone), an Nec-1 analog, has been protective against OGD/R-induced cell damage in cortical neurons, HT-22 cells and astrocytes [97]. Unfortunately, there are limited studies from OGD/R models where Nec-1 was protective, and in parallel, parameters of oxidative stress were measured. One example is the study in RGC-5 cells, where a necroptosis inhibitor, RIP1-inhibitory compound (RIC), reduced the OGD-induced damage and this effect was associated with a decrease in necroptosis markers (PI-positive cells, pRIP1), attenuated mitochondrial superoxide level, and restored mitochondrial polarization [98]. This directly proved that RIP1 inhibition could act on mitochondria and suppress ROS generation after OGD/R in retina cells [98]. In another study in mouse BV2 microglia cells, Nec-1 partially reversed the OGD-induced cell damage, which was associated with improved mitochondrial function, attenuated ROS level, decreased apoptosis rate, inhibited inflammasome activation (NLPR3, ASC, caspase-1), reduced inflammatory mediators (TNFα, IL-1β and MMP-9) and increased TGFβ level [99]. The above-mentioned effects of Nec-1 were comparable to those of recombinant human thioredoxin-1 (rhTrx-1), which has been shown in molecular docking studies to inhibit RIP1 kinase. Moreover, rhTrx-1 attenuated the increased OGD cell damage rate observed in co-culture of HT-22 with BV2 cells when compared to neuronal cell culture, suggesting a predominant role of microglial RIP1 in OGD-induced necroptosis in these cellular models [99]. Another OGD study showed a critical role of necroptosis in regulating ROS by knockdown RIP1 expression in BV2 cells with or without PGRN (progranulin) silence [100]. Nec-1 or PGRB overexpression abrogated the OGD-promoted expression of pRIP1, pRIP3 and pMLKL, which was associated with attenuation of ROS. Moreover, PGRN silencing significantly promoted OGD-induced ROS accumulation, which was markedly abrogated by RIP1 silencing or Nec-1 pretreatment [100]. In another model, in 12 DIV spinal cord neurons exposed to OGD, Nec-1 was at least partially protective, and this effect was associated with a decrease in RIP1 and pRIP3, increase in ATP and MMP (mitochondrial membrane potential) level, attenuation of oxidative stress (ROS and MDA) and increase in antioxidant capacity (SOD and GSH) [101].

All the above data suggest that in the OGD/R neuronal cell model, there is an interplay between oxidative stress and necroptosis and possibly also with other programmed cell death pathways, such as apoptosis or autophagy, suggesting that combined treatment with compounds targeting particular targets is fully justified.

### 4.4. Neuroprotective Effects of Necroptosis Inhibitors in In Vitro Intracerebral Hemorrhage Models

The mechanisms of intracerebral hemorrhage (ICH)-induced neuronal cell injury include oxidative stress, neuroinflammation, and apoptotic and non-apoptotic forms of programmed cell death. Iron, hemoglobin and hemin (oxidized form of heme) are used in neuronal or glia cellular models to study the mechanisms and therapeutic interventions against ICH [102,103]. Although in vitro studies investigating the involvement of necroptosis in ICH are rather limited, it has been shown that cell iron overload (exposure to ferrous chloride) evokes neurotoxicity in 8 DIV primary cortical neurons, which is reduced by Nec-1 in the presence of the caspase inhibitor z-VAD-fmk. These authors indicated that inhibiting both apoptosis and necroptosis would be the superior strategy for neuroprotection in ICH [102]. Another study showed that Nec-1, the ferroptosis inhibitor (ferrostatin-1), iron chelator (deferoxamine) and antioxidants (NAC, Trolox), but not Nec-1i or z-VAD-fmk partially reduced the neuronal cell damage induced by hemoglobin or hemin in three DIV primary mouse cortical neurons. Interestingly, Nec-1 and ferrostatin-1 were beneficial when given up to 8 h after induction of cell damage, whereas the therapeutic window for deferoxamine and NAC was shorter (2 and 4 h, respectively). The Nec-1-mediated protection in these cellular models was associated with the inhibition of RIP1 [103]. In another study, in HT-22 cells exposed to hemin, pretreatment with Nec-1 but not z-VAD-fmk partially reduced the extent of cell damage via attenuation of mitochondrial ROS level [104]. Altogether, all these data indirectly show the link between oxidative stress and various cell death programs including necroptosis in cellular ICH models (Table 1).

### 4.5. Neuroprotective Effects of Necroptosis Inhibitors in In Vitro Models of Parkinson’s Disease

The most significant pathological feature of PD is the progressive loss of dopaminergic neurons in the pars compacta of the substantia nigra (SNpc). In addition, PD has been associated with neurodegeneration of other neuronal cell types, including olfactory, cortical and autonomic peripheral neurons. All these processes contribute to motor, cognitive, psychiatric and peripheral symptoms in this chorea. Mitochondrial dysfunction and increased ROS level are thought to be the main causes of neuronal death in PD [105]. Cellular models of PD are mainly based on neuronal-like cell lines with dopaminergic phenotype (PC12 or SH-SY5Y cells) and primary mesencephalic neurons, which are damaged by dopaminergic neurotoxins, such as 6-hydroxydopamine (6-OHDA), 1-methyl-4-phenylpyridinium (MPP+), rotenone or paraquat [106,107]. Necroptosis has been suggested to be involved in the death process of dopaminergic neurons in PD [108]. It has been shown that pretreatment with Nec-1 (5–30 μM) partially prevented the 6-OHDA-induced cell damage in rat PC12 cells, which was accompanied by increased MMP, reduced expression of LC3-II and cathepsin B and increased Bcl-2. However, the higher concentrations of Nec-1 (60–90 μM) potentiated cell death induced by 6-OHDA [108]. In another study, it was shown that Nec-1s delayed 6-OHDA-induced axonal degeneration in seven DIV rat mesencephalic and cortical neurons without attenuation of DNA fragmentation evoked by this neurotoxin [105]. It confirms previous data obtained in a Glu-model of neuronal cell damage suggesting the co-activation of two cell death mechanisms in different neuronal compartments [84]. Moreover, in human UN- and RA-SH-SY5Y cells, Nec-1 was found to be protective against 6-OHDA-evoked cell damage, and this effect was similar to protection mediated by NAC and not changed after combined treatment with the caspase-3 inhibitor Ac-DEVD-CHO [46].

There are inconsistent data regarding the effects of Nec-1 in the MPP+ model of PD. In UN-SH-SY5Y cells, pretreatment with Nec-1 was not protective against MPP+-evoked cell damage, but it reduced protection mediated by group III metabotropic glutamate receptor agonists [109]. It indicates that the combined treatment with Nec-1 and another neuroprotectant not always results in a beneficial effect. Of note is that, in this experiment, the cell damage was partially inhibited by a caspase-3 inhibitor (Ac-DEVD-CHO), suggesting a predominant role of apoptosis in this cellular model of MPP+-induced cell damage [109]. In another study, Ito et al. [110] showed that Nec-1 and Nec-1i but not z-VAD-fmk prevented MPP+ induced RA-SH-SY5Y cell damage. Moreover, ferrostatin-1 and DFO also diminished the MPP+-induced toxicity, suggesting an interplay between necroptosis and ferroptosis in this model of neuronal cell damage and pointed to RIP1-independent mechanism of Nec-1 protective action. The mechanisms of Nec-1 mediated protection in this model were postulated to include the increase in MMP and intracellular ATP level, and attenuation of lipid peroxidation [110]. Another study showed that RIP3−/− mouse cortical neurons were less sensitive to MPP+−induced cytotoxicity, and this effect was associated with a lower level of caspase-3 activity and Bax/Bcl-2 ratio when compared to the wild-type cells [71]. To model neuroinflammation at the cellular level, PC12 cells were exposed to conditioned medium from LPS-treated mixed glia cells (high level of TNFα), which evoked cell damage via necroptotic mechanism (increased RIP1). Although in this model, pretreatment with Nec-1 was not protective, it synergized with z-VAD-fmk and significantly attenuated the PC12 damage induced by these neuroinflammatory factors present in conditioned medium from glial cells [48]. However, in mouse microglia cells (BV-2 or N9 cell lines, primary microglia) exposed to z-VAD-fmk and/or LPS and/or BV6 (SMAC mimetic), Nec-1 abolished cell damage induced by these factors, and this effect was associated with the inhibition of necroptotic factors (RIP1/RIP3/MLKL) and reduction in pJNK/JNK level [111]. Although Nec-1 was demonstrated to inhibit necroptosis induced by rotenone in SH-SY5Y cells measured by decreased level of pMLKL, it did not reduce but even exaggerated the Rot-evoked cell damage [112]. Additionally, in another neuronal cell type, namely RGC-5 cells, Nec-1 did not attenuate rotenone toxicity, whereas inhibitors of JNK and p38 pathways exerted beneficial effects in this model [113]. Altogether, the above studies suggest that efficacy of Nec-1 in protecting neuronal cells in in vitro PD models strongly depends on the type of neurotoxic agents and point to a minor role of necroptosis as compared to apoptosis in the mechanism of this type of cell injury. Nevertheless, some of these studies showed a link between oxidative stress parameters and necroptosis induction [46,110].

### 4.6. Neuroprotective Effects of Necroptosis Inhibitors in Other Cellular Models

Although oxidative stress and necroptosis have been evidenced to be involved in the pathogenesis of AD [114], and although there are some data (Table 1) showing neuroprotective effects of Nec-1 in cellular models of this pathology [115,116,117,118], there is a lack of studies showing a direct link between oxidative stress and necroptosis in this type of neuronal degeneration.

With respect to neuronal cells, the activation of Akt signaling usually contributes to neuroprotection mediated by various synthetic or natural compounds [119]. However, in the classical necroptosis model (TNFα+z-VAD-fmk) in neuronal HT-22 cells, an unexpected role for Akt and mTOR downstream of RIP1 activation has been found [120]. In this model, Nec-1 but not Nec-1i attenuated necroptotic cell damage by inhibition of Akt and mTOR phosphorylation, reduction in mitochondrial ROS, and prevention of RIP1-RIP3-pAkt complex assembly. These data extended the range of upstream regulators of necroptosis to Akt and mTOR pathways, which is also associated with the regulation of mitochondrial ROS [120]. Additionally, Nec-1 completely attenuated the astrocyte cell death evoked by TNFα+LPS+z-VAD-fmk via mechanisms engaging reduction of necroptotic proteins (RIP3, MLKL, HMGB1), attenuation of ROS and increase in ATP level [121].

Moreover, necroptosis has been postulated to be involved in white and blue-light-induced death of RGC-5 cells and pretreatment with Nec-1 but not with z-VAD-fmk reduced cell damage induced by these physical factors [122,123]. The neuroprotective effect of Nec-1 against blue light-evoked cell damage was not associated with the prevention of the cleavage of AIF but with the attenuation of heme-oxygenase-1 (HO-1) mRNA. Since RIP1 and RIP3 gene silencing had no effect on HO-1, it has thus been suggested that Nec-1, via additional (apart from RIP1/RIP3) mechanisms, reduced oxidative stress evoked by cell exposure to blue light [122]. Although plant-derived flavonoids are regarded as potential neuroprotectans due to their antioxidant properties, at higher concentrations, these compounds may also reveal undesired prooxidative effects. Along this line of research, the flavonoids myricetin and, at higher concentrations (100–200 μM), quercetin were reported to decrease the viability of the primary cultures of human retinal pigment epithelial cells by triggering cellular necroptosis. Nec-1 and calpain inhibitors but not Nec-1i or caspase inhibitor (Ac-DEVD-CHO) inhibited neuronal cell damage induced by these phytochemicals [124]. All the above data support promising neuroprotective effects of Nec-1 or other inhibitors of RIP1 in retina degeneration, which occurs in glaucoma and diabetic retinopathy, which also implicates oxidative stress regulation. Additionally, in mechanically injured neurons, Nec-1 could be protective, as shown in TBI in vitro model traumatic neuronal injury (TNI). Damage to 14–16 DIV cortical neurons by mechanical scratches caused an increase in immediate-early gene Arc (activity-regulated cytoskeleton-associated protein) expression, intracellular Ca^2+^ level and expression of ER stress markers (Grp78, CHOP, caspase-12) and RIP1. Downregulation of Arc by specific shRNA augmented all these parameters and increased cell death in TNI-exposed cells. Nec-1 and the mGluR1 antagonist AIDA but not Nec-1i partially prevented the detrimental effects of TNI in Arc-silenced neurons [125]. These data suggest an interplay between Arc, ER and RIP1 and possibly with ROS (indirectly linked with increased Ca^2+^ level), which could be crucial for endogenous protection after acute neuronal injuries.

**Table 1:** Neuroprotective effects of necroptosis inhibitors in in vitro models directly or indirectly connected with oxidative stress.

Model	Inducer	Cell Type	Necroptosis Inhibitor	Ref.
Oxidative stress	0.1–20 mM BSO 50–400 uM H_2_O_2_ + 200 uM BSO 2 mM H_2_O_2_ 0.25 mM H_2_O_2_ 0.5 mM H_2_O_2_ 5 mM Glu 5 mM Glu 4 mM Glu 3 mM Glu 160 mM Glu 5 mM Glu/BSO 5 mM Glu/BSO 100 uM arachidonic acid cysteine deprivation 5 mM BSO 100 and 400 uM H_2_O_2_	mouse HT-22 cells human SK-N-SH cells mouse HT-22 cells human UN-SH-SY5Y cells human RA-SH-SY5Y cells mouse HT-22 cells mouse HT-22 cells mouse HT-22 cells mouse HT-22 cells human RA-SH-SY5Y cells mouse RGC-5 cells mouse RGC-5 cells 7–9 DIV rat OPCs 7–9 DIV rat OPCs 7–9 DIV rat OPCs 7–9 DIV rat OPCs	Nec-1 10 uM-complete Nec-1 40 uM-complete Nec-1 10–40 uM-partial 10–40 uM-partial 20–40 uM-partial Nec-1 25–100 uM-complete Nec-1 10 uM-complete Nec- 1 50 uM-complete Nec-1 10–40 uM-partial Nec-1 50 uM-complete Nec-1 50–100 uM-partial Nec-1 25–50 uM-partial Nec-1 20 uM-complete Nec-1 20 uM-complete Nec-1 20 uM-complete Nec-1 no protection (20 uM)	[73] [74] [46] [46] [46] [73] [77] [76] [46] [78] [79] [80] [81] [81] [81] [81]
Excitotoxicity	100 uM NMDA 20 uM Glu	10–12 DIV rat cx neurons 7–15 DIV rat hip. neurons	Nec-1 30–100 uM-partial Nec-1 100 uM	[83] [84]
Ischemia/Hypoxia	1 h OGD/24 h R 2 h OGD/24 h R 2 h OGD/3 h R 2 h OGD/0–8 h R 3–12 h OGD 3 h OGD/R 48 h 48 h hypoxia 8 h OGD/0–24 h R 4 h OGD/24 h R 8 h OGD/6–12 h R 3–12 h OGD 6–12 h OGD/ 24h R 3 h OGD + zVAD/24 h R CM from OGD + zVAD neu 12 OGD/4–48 h R 2 h OGD/24 h R 12 h OGD 6 h OGD/R 24 h 9 h OGD 4 h OGD/24 h R 4 h OGD/24 h R 6–24 h OGD/R 2 h OGD/2 h re-oxyg	10 DIV mouse cx neurons 15 DIV rat hip. neurons 4 DIV rat cx neurons 8 DIV rat cx neurons 5–8 DIV rat cx neurons 7 DIV rat cx neurons 7 DIV rat cx neurons rat PC12 cells 2 DIV mouse RGCs mouse RGC-5 cells rat cx astrocytes mice cx astrocytes 10 DIV primary neurons mouse primary microglia cells mouse N9 cells 12 DIV mouse OPCs rat cx neurons/astrocytes; HT-22 cells rat cx neurons/astrocytes; HT-22 cells mouse RGC-5 cells mouse BV2 cells mouse co-culture HT-22+BV2 cells mouse BV2 cells mouse spinal cord neurons	Nec-1 25 uM-partial Nec-1 20 uM-partial Nec-1 2 uL of 1%-partial Nec-1 20 uM-partial Nec-1 1–100 uM-partial Nec-1 25 uM-partial Nec-1 6.25–50 uM-partial Nec-1 20 uM-partial Nec-1 20 uM-partial Nec-1 10 uM-partial Nec-1 1–100 uM-partial Nec-1 10 uM-partial Nec-1 no protection (20 uM) Nec-1 20 uM-complete Nec-1 20 uM-partial Nec-1 20 uM-partial DTIO 10 uM DTIO 10 uM RIC 3–20 uM Nec-1 20 uM-partial, rhTrx-1 Nec-1 20 uM-partial, rhTrx-1 Nec-1 30 uM-partial, PGRN Nec-1 20–50 uM-partial	[85] [86] [87] [88] [89] [90] [90] [88] [91] [92] [89] [93] [94] [94] [95] [96] [97] [97] [98] [99] [99] [100] [101]
Intracerebral hemorrhage	100 uM ferrus chloride 100 uM hemin 1.5 uM hemoglobin 50 uM hemin	8 DIV mouse cx neurons 3 DIV mouse cx neurons 3 DIV mouse cx neurons mouse HT-22 cells	Nec-1 30 uM-partial Nec-1 50–100 uM-partial Nec-1 50–100 uM-partial Nec-1 30 uM-partial	[102] [103] [103] [104]
PD-like models	100 uM 6-OHDA 40 uM 6-OHDA 40 uM 6-OHDA 100 uM 6-OHDA 200 uM 6-OHDA 1 mM MPP+ 5 mM MPP+ 15–25 uM MPP+ CM from LPS-glia cells z-VAD-fmk/LPS/BV6 100 nM rotenonec 10 uM rotenone	rat PC12 cells 7 DIV rat mes. neurons 7 DIV rat cx neurons human UN-SH-SY5Y cells human RA-SH-SY5Y cells human UN-SH-SY5Y cells human RA-SH-SY5Y cells 7 DIV mouse cx neurons rat PC12 cells mouse BV2 and N9 microglia cells human SH-SY5Y cells mouse RGC-5 cells	Nec-1 5–30 uM-partial Nec-1s 30 uM Nec-1s 30 uM Nec-1 20–40 uM-partial Nec-1 40 uM-partial Nec-1 no protection (10 uM) Nec-1 and Nec-1i 20 uM-partial RIP3−/− cells Nec-1 no protection (20 uM) Nec-1 30 uM-complete Nec-1 no protection (20–30 uM) Nec-1 no protection (50–200 uM)	[108] [105] [105] [46] [46] [109] [110] [71] [48] [111] [112] [113]
AD-like models	A-beta aggregation 10 uM A-beta1–42 10 uM A-beta1–42 2–8 mM Aluminum 2 mM Aluminum	Human MC65 cells (TC-control) Mouse HT-22 cells Mouse BV2 cells Human SH-SY5Y cells 5 DIV mouse cx neurons	Nec-1 30–100 uM-complete Nec-1 50–100 uM-complete Nec-1 50–100 uM-complete Nec-1 60–90 uM-complete Nec-1 60–90 uM-complete	[115] [116] [116] [117] [118]
Other models	TNFa + zVAD TNFa + CHX + zVAD CM from TNFa + LPS + zVAD- astrocytes blue light (250 lx) light (1000 lx) sodium azide 200 uM myricetin or quercetin mechanical injury	mouse HT-22 cells mouse spinal cord astrocytes 7 DIV mouse spinal cord neurons mouse RGC-5 cells mouse RGC-5 cells mouse RGC-5 cells human retina epithelial cells 14–16 DIV mouse cx neurons	Nec-1 30 uM-complete Nec-1 20 uM-complete Nec-1 20 uM-complete Nec-1 50 uM-partial Nec-1 25–50 uM-partial Nec-1 no protection (25–50 uM) Nec-1 30 uM-complete Nec-1 100 uM-partial	[120] [121] [121] [122] [123] [123] [124] [125]

6-OHDA—6-hydroxydopamine; A-beta—beta-amyloid; BSO—buthionine sulfoximine; CM—conditioned medium; cx—cortical; DFO—deferoxamine; DIV—day in vitro; ER—endoplasmic reticulum; Glu—glutamate; hip.—hippocampal; Nec-1—necrostatin-1; mes.—mesencephalic; MPP+—1-methyl-4-phenylpyridinium; OGD/R—oxygen-glucose deprivation/re-oxygenation; OPCs—oligodendrocyte precursor cells; PGRN—progranulin; RA—retinoic acid differentiated; RGCs—retinal ganglion cells; RIC-RIP1 inhibitory compound; RIP3—receptor interacting protein 3; rhTrx-1—recombinant human thioredoxin-1; ROS—reactive oxygen species; TC—tetracycline.

## 5. In Vivo Studies Linking Oxidative Stress and Necroptosis in Relation to Neurodegenerative Diseases

In contrast to a number of in vitro studies, there are only a few studies on animal models of neurodegenerative diseases, indicating an association between oxidative stress and necroptosis. Acute brain damages, such as ischemic or hemorrhagic stroke, neonatal hypoxia/ischemia (NHI), TBI and SCI lead to increased ROS production, which evokes secondary damage via activation of the NLRP3 inflammasome and increased production of pro-inflammatory cytokines (IL-1β, TNFα or IL-6) [59,126]. Thus, apart from animal studies that in parallel measured both oxidative stress and necroptosis parameters after treatment with necroptosis inhibitors, reports showing the impact of necroptosis inhibitors on neuroinflammation markers also provide indirect evidence for a close relationship between oxidative stress and necroptosis (Table 2).

Direct evidence for the association between necroptosis and oxidative stress was provided by Jiao et al. [99], who in the transient MCAO (middle cerebral artery occlusion) model in mice showed neuroprotective effects of rhTrx-1, an antioxidant and anti-inflammatory agent [127]. This effect was associated with attenuation of ischemia-induced RIP1 expression and occurrence of Iba1+/RIP1+cells, pointing to an involvement of necroptosis and showing that microglia cells are an important player in the rhTrx-mediated protection. Other reports from the ischemia field that indirectly link necroptosis with oxidative stress showed neuroprotection mediated by Nec-1 or other necroptosis inhibitors, and this effect was accompanied by the attenuation of pro-inflammatory markers. In particular, Li et al. [97] showed that DTIO, a novel analog of Nec-1, reduced brain damage and attenuated neurological deficits after MCAO in mice and rats, and this beneficial effect was associated with a reduction in pro-inflammatory cytokine levels (TNFα, IL-1β, IL-6) in the cerebral cortex. Another study using the rat MCAO model revealed that Nec-1 reduced infract volume and attenuated ischemia-related neurological deficits, and these effects were accompanied by a decrease in necroptotic (p-RIP1+neurons, p-RIP1, RIP3, MLKL, pMLKL) and inflammatory (IL-1β) markers [128].

Regarding NHI, the first study to show neuroprotection by Nec-1 in this brain damage model also demonstrated that apart from the inhibition of RIP1-RIP3 complex formation, this compound attenuated protein oxidation, NFκB activity and inflammatory response (TNFα, IL-1β, IL-6, IL-12) [129]. In their next study, the same authors found that neuroprotection mediated by Nec-1 in this model of brain damage was associated with reduced glutathione oxidation, decreased HIF-1α/BNIP3 level, increased mitochondrial complex-I activity and ATP levels and attenuation of ER stress markers (PERK and eIF2α phosphorylation, GADD43 and unconventional XBP-1 splicing) [130,131].

With respect to brain damage induced by intracranial hemorrhages, some studies provided further evidence for an indirect link between necroptosis and oxidative stress, pointing to a pivotal role of inhibition of neuroinflammation in the mechanism of neuroprotective effects of Nec-1 in the SAH (subarachnoid hemorrhage) rat models. Chen et al. [132] demonstrated that during brain injury evoked by endovascular perforation, there was an increase in expression of necroptosis proteins (RIP1, RIP3 and MLKL), activation of NFκB and increased production of pro-inflammatory cytokines (TNFα and IL-1β). These changes were attenuated by Nec-1, which also partially ameliorated brain swelling, reduced the lesion volume, reduced the number of PI-positive cells and improved neurological outcomes. In another study using also the SAH rat model, it was demonstrated that Nec-1 or mitochondrial division inhibitor (Mdivi-1) alleviated both brain edema and neurological deficits and in parallel reduced necroptosis (RIP1, RIP3), mitochondrial damage (phosphorylated DRP1) and NLRP3 inflammasome activation in the insulted brain tissue. Moreover, Mdivi-1 also decreased the injury-induced ROS production [133].

Regarding TBI, it was shown that the necroptosis inhibitor Nec-1 and melatonin (an antioxidant) significantly reduced morphological changes (cortical lesion, brain edema) and improved neurological functions (motor ability and learning and memory) in the controlled cortical impact (CCI) mice model. Furthermore, both compounds decreased necroptotic markers (RIP1, RIP3 and MLKL) and the number of TUNEL-positive cells, decreased HMGB1 and RAGE levels, ameliorated NFκB activation and neuroinflammatory protein (IL-1β, IL-6 and NLRP3) levels [134]. In the animal SCI models, Nec-1 was also protective via attenuation of oxidative stress parameters, necroptosis and neuroinflammation. In the rat SCI model, Nec-1 downsized the lesion and reduced the number of TUNEL-positive cells, decreased levels of necroptotic (RIP1, RIP3 and MLKL) and apoptotic (caspase-3, Bax/Bcl-2) markers, diminished oxidative stress (ROS, MDA), increased antioxidant enzymes (SOD) and inhibited pro-inflammatory cytokine release (TNFα, IL1β and IL-6) [135]. In the next study, the same research group evidenced that Nec-1 improved mitochondrial functions by decreasing mitochondrial Ca^2+^ accumulation, preserving MMP and increasing ATP production. Moreover, the necroptosis inhibitor promoted the activity of mitochondrial complex I, inhibited cytochrome c release and helped to maintain the normal mitochondrial ultrastructure and modulation of mitochondrial biogenesis [136]. Finally, the beneficial effects of Nec-1, hinting at a link between necroptosis and oxidative stress, were confirmed in the mouse SCI model, where Nec-1 improved locomotor function and reduced spinal cord edema, decreased RIP1, pRIP3, ROS and MDA and increased ATP, MMP, SOD and GSH levels in the injured spinal cord tissue [101]. Similar protective efficacy and biochemical mechanisms of action (except for the influence on RIP1) were found for the RIP3 inhibitor GSK’872 [101].

Additionally, studies in animal retina degeneration models (P23H rhodopsin mutant rats and mice) showed that necroptosis (RIP1, RIP3) and neuroinflammation (NFκB, inflammasome, microglia activation) parameters were increased and that Nec-1 could attenuate these changes including ROS inhibition. Interestingly, these neuroprotective effects of Nec-1 were superior to the effect mediated by the potent and well-recognized antioxidant NAC [137]. Another study showed that the combination of Nec-1 with the apoptosis inhibitor z-VAD-fmk was protective against mechanical retina injury, as evidenced by the decreased number of TUNEL-positive cells, increased outer nuclear layer (ONL) thickness, reduced oxidative stress, prevention of AIF translocation and attenuated number of CD11-positive microglia/macrophages. Similar effects were observed in RIP3−/− mice, which appeared to be protected against this type of retina injury [138].

As far as chronic neurodegenerative diseases are concerned, there are so far rather limited data showing the interplay between oxidative stress, necroptosis and/or neuroinflammation. Regarding AD, in the model of spatial memory impairment in rats induced by neuroinflammation (LPS), Nec-1 attenuated cognitive deficits and reduced the level of necroptotic (RIP1, RIP3), apoptotic (Bax/Bcl2, caspase-8 and caspase-3) and oxidative stress (MDA) parameters and increased antioxidant defense (SOD, GSH and catalase) in the hippocampus and frontal cortex [139]. Moreover, in the rat model of prediabetes, which is characterized by cognitive decline and brain pathology, Nec-1 improved cognitive function, synaptic plasticity and brain mitochondrial function and reduced hyperphosphorylated Tau formation. Additionally, in this model, Nec-1 attenuated necroptosis (pRIP1, RIP3), oxidative stress and microglia activation [140]. Additionally, it has been shown that Nec-1 could be efficient in improving cognitive performance in aged animals, and these effects were accompanied by decreased RIP1 level, improved synaptic plasticity and alleviated pro-inflammatory cytokine (IL-1α, IL-1β and TNFα) contents [141].

Regarding PD models, Iannielli et al. [142] showed that three-week treatment of mice with Nec-1 and Nec-1s provided protection against MPTP-induced dopamine neuron loss in the substantia nigra pars compacta (SNpc), and this protective effect was associated with reduced intracellular oxidative stress levels (4-hydroxynonenal). Another study showed that Nec-1-treated WT mice and RIP3−/− or MLKL−/− mice were protected against MPTP and the treatment also decreased the level of pro-inflammatory cytokines (TNFα, IL-1β and IL-6) [143].

Among other types of nerve injury that could link necroptosis with neuroinflammation and indirectly with oxidative stress are peripheral nerve injury or multiple sclerosis (MS). Liang et al. [144] found that in a rat sciatic nerve chronic constriction injury model, Nec-1 reduced behavioral symptoms, and it was associated with attenuation of necroptosis-related protein levels (RIP1, RIP3), reduction in the number of PI-positive cells, inhibition of NFκB activity and decreased level of pro-inflammatory cytokines (TNFα, IL-1β) and substance P. Finally, in experimental autoimmune encephalomyelitis (EAE) Nec-1 attenuated lesions in the spinal cord tissue, diminished production of pro-inflammatory cytokines (TNFα, IL-1β and INFγ), increased pDrp1/Drp1 and reduced necroptotic (pMLKL/MLKL) and apoptotic (Bax/Bcl-2, Bim) markers [145].

It should be stressed that necroptosis does not appear to be important for all types of neurodegeneration. For instance, the study by Wang et al. [146] using mutant SOD1G93A mice clearly demonstrated that necroptosis was unlikely to play a pathogenic role in ALS. Their data challenged the proposal that inhibition of necroptotic signaling would be therapeutic for ALS, because RIP1 elevation was observed only in the late phase of the disease, and the RIP3-MLKL signaling axis was absent in the affected motor neurons and inflammatory glial cells. Their findings suggested that alternative cell-death pathways, aside from apoptosis and necroptosis, may be crucial to astrocyte-mediated motor neuron death in ALS [146]. Collectively, results of the currently limited number of in vivo studies suggest that inhibition of oxidative stress significantly contributes to neuroprotective effects of necroptosis inhibitors. Furthermore, the link between oxidative stress and necroptosis-signaling cell death pathways seems to be of special importance in the pathomechanism of neurodegenerations strongly associated with neuroinflammation.

**Table 2:** Animal models of neurodegeneration linking necroptosis with oxidative stress and/or neuroinflammation.

Disease	Animal Model	Neuroprotective Compound	Ref.
Stroke	MCAO/R in C57 Bl mice MCAO/R in ICR mice MCAO/R in SD rats MCAO/R in SD rats	rhTrx1 10 mg/kg *i.v.* DTIO 1–10 mg/kg *i.v.* DTIO 10 mg/kg *i.v.* + *i.p.* for 7 or 28 d. Nec-1 1.5 uL/20 mM *i.c.v.*	[99] [97] [97] [128]
Neonatal hypoxia/ischemia	Hypoxia in C57Bl mice Hypoxia in C57Bl mice Hypoxia in C57Bl mice	Nec-1 0.1 uL/80 uM *i.c.v*. Nec-1 0.1 uL/80 uM *i.c.v.* Nec-1 0.1 uL/80 uM *i.c.v*.	[129] [130] [131]
Hemorrhagic stroke	SAH in SD rats SAH in SD rats	Nec-1 200 ug *i.c.v.* Nec-1 10.5 mg/kg *i.p*.; Mdivi-1 3.6 mg/kg *i.p*.	[132] [133]
TBI/SCI	CCI in SD rats laminectomy/T10 in SD rats laminectomy/T10 in SD rats laminectomy/Th6–7 in C57Bl mice	Nec-1 6 uL/25 mM *i.c.v*.; melatonin 20 mg/kg *i.p*. Nec-1 1–50 ug *i.t.* Nec-1 25 ug *i.t.* Nec-1 5 mg/kg *i.p*.; GSK’872 2 mg/kg *i.p*.	[134] [135] [136] [101]
Retina injury	Tg P23H rhodopsin rat and mice mutants retinal detachment in Norway rats and in C57BL WT and RIP3−/− mice	Nec-1 15 mg/kg/day *s.c.* NAC 150 mg/kg/day *s.c*. from PD21 to PD120 400 uM Nec-1 + 300 uM z-VAD-fmk *i.r*. in WT RIP3−/−	[137] [138]
AD/aging	LPS i.c.v. in Wistar rats Hight fat diet in rats D-galactose+hepatoctomy in C57Bl mice	Nec-1 10 uM *i.c.v.* Nec-1 1.65 mg/kg/day *s.c*. from 13 to 21 week Nec-1 6.25 mg/kg *i.p*.	[139] [140] [141]
PD	MPTP in C57Bl mice MPTP in C57Bl WT, RIP3−/− and MLKL−/− mice	Nec-1 1 ug/day *i.c.v*.; Nec-1s 10 mg/kg/day *i.p*.from 3–21 days Nec-1 1.65 mg/kg/day *i.p*. up to 21 days; RIP3−/− and MLKL−/−	[142] [143]
Other	sciatic nerve chronic constriction (CCI) in SD rats EAE in C57Bl	Nec-1 0.2–0.4 mg/kg/day *i.p*. for 21 days Nec-1 1.65 mg/kg *i.t*. from day 2 every 3 days for 15 days	[144] [145]

AD—Alzheimer’s disease; EAE—experimental autoimmune encephalomyelitis; *i.c.v.*—intracerebroventrical; *i.p.*—intraperitoneally; *i.r.*—intraretinal; *i.t.*—intrathecal; *i.v.*—intravenous; LPS—liposaccharide; MCAO/R—middle cerebral artery occlusion/reperfusion; MLKL—mixed lineage kinase-like protein kinase; MPTP—1-methyl-4-phenyl-1,2,3,6-tetrahydropyridine; Nec-1—necrostatin-1; PD—Parkinson’s disease; rhTrx-1—recombinant human thioredoxin-1; RIP3—receptor interacting protein 3; rhTrx-1—recombinant human thioredoxin-1; SAH—subarachnoid hemorrhage; *s.c.*—subcutaneous; SCI—spinal cord injury; SD—Sprague Dawley; TBI—traumatic brain injury; WT—wild type.

## 6. Multipotential Neuroprotectants for Future Treatment of Acute and Chronic Neurodegenerative Diseases

Recent decades have witnessed remarkable progress in elucidating the molecular mechanisms of neuronal death in acute and chronic neurodegenerative diseases. It has been firmly established that the pathomechanism of neuronal death may involve excitotoxicity and dysregulation in calcium homeostasis, and consequently, perturbations in mitochondria function, activation of proteolytic enzymes, increased production of deleterious ROS and decreased cell defense system function. Cytoskeletal disruptions, protein aggregation, deficiency of neurotrophic factors and neuroinflammation are also detected in the majority of NDs. Moreover, the presence of free iron and other transition metals could also facilitate oxidative-stress-related neurodegeneration in specific brain areas. Accordingly, putative neuroprotectants are being searched among substances that could delay the degeneration of neurons by interfering at particular stages of neuronal cell death to inhibit excitotoxicity, apoptosis, ROS production, mitochondrial disruption, necroptosis or inflammation. Promising candidates for antiexcitotoxic strategies can be found among antagonists of NMDA and AMPA ionotropic glutamate receptors, allosteric modulators of metabotropic mGluR receptors and other agents that prevent excessive glutamate release, e.g., riluzole. On the other hand, antiapoptotic agents proposed to be used as neuroprotectants comprise mainly inhibitors of caspase-3, which is an effector caspase in both intracellular and extramitochondrial cell death pathways. Other methods to block apoptosis consider inhibition of the c-Jun-N-terminal protein kinase (JNK) pathway, the AMP-activated kinase (AMPK), which is the main sensor of intracellular energetic balance, and calpain inhibitors. Other experimental approaches focus on inducing the expression of antiapoptotic Bcl-2 family members. Necroptosis can be prevented by inhibitors of RIP1, such as Nec-1. Regarding antioxidant approaches, among the best known putative neuroprotectants and free-radical-neutralizing agents are polyphenols, e.g., quercetin, vitamins E, lazaroids, estrogens, tirilazad, ebselen, edaravone and NXY-059. Besides antioxidants, agents restoring mitochondrial function or preventing the permeabilization of their membranes, such as coenzyme Q, creatine, inhibitors of the permeability transition pore complex (in particular ligands of cyclophilin D), openers of mitochondrial ATP-sensitive or Ca^2+^-activated K^+^ channels, are also tested. In order to prevent deleterious effects of neuroinflammation, some substances that prevent activation of microglia and astrocytes, formation of superoxide radicals and nitric oxide and production of proinflammatory cytokines such as TNFα are vigorously investigated. Close attention has been devoted to endogenous neuroprotective agents that help to maintain neuronal homeostasis under pathological conditions. The list of putative endogenous neuroprotective agents includes substances with diverse chemical structures, such as adenosine, neurotrophins, chaperone proteins, amino acids (kynurenic acid, taurine), neuropeptides (opioid peptides, thyreoliberine, neuropeptide Y, galanin, VIP/PACAP), hormones (estrogens) and some neurosteroids (allopregnanolone, dehydroepiandrosterone).

A large number of plant-derived and synthetic antioxidants have been extensively tested as putative neuroprotectants in preclinical and clinical studies. The general conclusion from these studies was that compounds with a single mechanism of action typically showed limited neuroprotective potential. Therefore, it seems that a more promising strategy is to search for compounds with multipotential action and/or to test combinations of agents that target various cellular death pathways. Natural compounds have long been considered as supplementary drugs in preventing age-related neurodegenerative diseases; however, their influence on particular cell death pathways has been only recently acknowledged. Specifically, preclinical studies showed that many herbs used in traditional Chinese medicine target multiple mechanisms of regulated cell death and, in combination, may exert synergistic effects on signaling pathways, thereby attenuating multiple aspects of ischemic neuronal injury [147]. For example, curcumin isolated from plants displayed pleiotropic neuroprotective activities in various in vitro and in vivo models. This compound was shown to attenuate ferrous chloride-induced necroptosis in primary cortical neurons and to decrease expression of RIP1 in a dose- and time-dependent manner [102]. Thus, the ability of curcumin to act as an antioxidant and chelator of free radicals inducing iron ions, along with the inhibition of RIP1-dependent necroptosis, may contribute to its efficacy in protecting neuronal cells against neurotoxic agents. Another natural antioxidant and anti-inflammatory compound, sulforaphane, was suggested to have neuroprotective effects in different NDs. Recently, it was demonstrated that sulforaphane indirectly suppressed microglia-mediated neuronal damage, as evidenced by inhibition of necroptosis and reduced expression of RIP3 and MLKL, and these effects involved p38, JNK and NFκB p65 but not ERK1/2 signaling pathways [148]. Another interesting plant-derived compound gallic acid (3,4,5-trihydroxybenzoic acid) was studied in intranigral LPS-induced neuroinflammation in rats. This compound not only attenuated inflammatory biomarkers but also prevented LPS-induced caspase 3 activation and increases in receptor-interacting protein kinase (RIP1 and RIP3) levels. Besides its ability to inhibit LPS-induced apoptosis and necroptosis in the nigrostriatal dopaminergic system of rat brain, gallic acid revealed strong antioxidant properties in vitro that are more potent than glutathione, though less potent than Trolox, in inhibiting the iron-induced lipid peroxidation. Collectively, these data suggest that gallic acid may be neuroprotective in ND [149]. Other researchers demonstrated that water-soluble extract from the culture medium of Ganoderma lucidum mycelia, which is a popular medicinal mushroom in China and Japan, suppressed ischemia-induced superoxide production, neuronal cell death, and biochemical and histological markers of apoptosis and necroptosis in type 2 diabetic KKAy mice [150]. Flavonoids, a large group of natural substances with variable phenolic structures, are well recognized for their antioxidant activity and preventing cell damage by scavenging ROS, but their effect on various cellular death signaling pathways have been only partially unraveled. Flavonoids, such as epicatechin, quercetin, genistein, daidzein not only are ROS scavengers and chelators of Fe^2+^ and Cu^2+^, but they are also modulators of cell signaling (phosphorylation, gene expression). Baicalein (a flavonoid of *Scutellaria baicalensis Georgi*) inhibited oxidative stress and attenuated neuroinflammation, apoptosis and necroptosis in the acrolein-induced model of neurodegeneration of nigrostriatal dopaminergic system. These data suggest that baicalein might be therapeutically useful for slowing PD progression [151].

Besides plant-derived compounds, other natural sources of putative neuroprotectants with multiple mechanisms of action, including both antioxidant and antinecroptotic properties, are being vigorously investigated. It is worth mentioning here that the glycoprotein progranulin is a precursor of multifunctional cysteine-rich proteins granulins, which are involved in cell cycle control, wound healing, inflammatory and carcinogenic processes, neuron development and neurodegeneration. It was found in the model of ischemic stroke that progranulin attenuated brain ischemia/reperfusion-induced oxidative damage partly via its regulatory effects on necroptosis. Furthermore, enhanced progranulin expression reduced brain injury by suppressing necroptosis and associated ROS production [100].

## 7. Conclusions

Despite the progress in understanding the molecular mechanisms of neuronal injury and the development of methods to prevent them in preclinical experiments, only a few neuroprotective substances are used in the clinic, and their efficiency in the treatment of stroke and neurodegenerations is not satisfactory. Since most putative neuroprotective drugs are giving hope based on preclinical studies’ lack of efficiency in clinical trials, attention should be focused on searching substances with multipotential action on neuronal cell death pathways or combining few drugs that possess neuroprotective activity. Such combinations may include, for example, antagonists of glutamate receptors with inhibitors of apoptosis, or with antioxidants and antinecroptopic agents. The present review summarized preclinical evidence for an interplay between oxidative stress and necroptosis and suggested the combined administration of antioxidants and necroptosis inhibitors as a supportive strategy in the treatment of acute and chronic neurodegenerative disorders. The main rationale for such an approach is the emerging key role of necroptosis in various pathologies, including inflammatory and neurodegenerative diseases, the pathomechanisms of which indisputably engage also oxidative stress. However, the preclinical data also revealed great complexity of interactions between various programmed cell death signaling pathways and their regulatory mechanisms, which have been only partially untangled. Furthermore, it should be stressed that available necroptosis inhibitors, as well as natural or synthetic antioxidants, possess unfavorable pharmacokinetic properties. Their action is time- and concentration-dependent and strongly depends on the model of neuronal damage and the stage of its development. Hence, it appears that further improvement of ND treatment outcomes may depend not only on a better understanding of the molecular mechanism of neuronal damage, but also on pharmacokinetic properties of the neuroprotective drugs, innovative delivery systems, e.g., nanocarriers, and on rigorously designed and conducted clinical trials.

## Abbreviations

AD—Alzheimer’s disease; AIF—Apoptosis Inducing Factor; ALS—Amyotrophic Lateral Sclerosis; BSO—buthionine sulfoximine; CAT—catalase; CNS—central nervous system; DAMPs—Damage-Associated Molecular Patterns; ER—endoplasmic reticulum; ETC—electron transfer chain; FADD—Fas-associated via death domain; GSH—glutathione; HMGB1- high-mobility group box 1 protein; HNE—4-hydroxynonenal; LPS—liposaccharide; MDA—malondialdehyde; MLKL—mixed lineage kinase-like protein kinase; MPP+ -1-methyl-4-phenylpyridinium; MPTP—1-methyl-4-phenyl-1,2,3,6-tetrahydropyridine; ND—neurodegenerative diseases; Nec-1—necrostatin 1, 5-(1H-Indol-3-ylmethyl)-(2-thio-3-methyl) hydantoin, methyl-thiohydantoin-tryptophan; NO—nitric oxide; NOXs—NADPH oxidases; NSA—necrosulfonamide; OGD/R—oxygen-glucose deprivation/re-oxygenation; PD—Parkinson’s disease; RIP1—receptor-interacting serine/threonine-protein kinase 1; RIP3—receptor-interacting serine/threonine-protein kinase 3; RNS—reactive nitrogen species; ROS—reactive oxygen species; SOD—superoxide dismutase.
